# Characteristics of Hepatitis B Virus Genotype and Sub-Genotype in Hepatocellular Cancer Patients in Vietnam

**DOI:** 10.3390/diagnostics12102393

**Published:** 2022-10-01

**Authors:** Phat Tan Ho, Mario Giosuè Balzanelli, Pietro Distratis, Rita Lazzaro, Duy Khanh Tran, Kieu C. D. Nguyen, Tri Minh Bui, Thinh Tien Nguyen, Son Truong Pham, Huy Song Dinh Nguyen, Vinh Thanh Tran, Toan Trong Ho, Gianna Dipalma, Francesco Inchingolo, Camelia Quek, Huong Thien Pham, Ciro Gargiulo Isacco, Luigi Santacroce, Van Hung Pham

**Affiliations:** 1 Cho Ray Hospital Ho Chi Minh Vietnam, Phat Tan Ho, Ho Chi Minh 749000, Vietnam; 2 SET-118, Department of Pre-Hospital and Emergency, SG Giuseppe Moscati Hospital, 74100 Taranto, Italy; 3 Nam Khoa Biotek, Ho Chi Minh 500000, Vietnam; 4 Environmental Engineering, Ho Chi Minh University of Technology, Ho Chi Minh 700000, Vietnam; 5 American Stem Cells Hospital Ho Chi Minh, Ho Chi Minh 700000, Vietnam; 6 108 Military Central Hospital, Hanoi 100000, Vietnam; 7 Western Sydney Local Health District, Sydney 2170, Australia; 8 School of Medicine, University of Bari “Aldo Moro”, 70124 Bari, Italy; 9 Faculty of Medicine and Health, The University of Sydney, Sydney 2050, Australia; 10 Multidisciplinary Clinic, University of Medicine Pham Ngoc Thach, Ho Chi Minh 700000, Vietnam; 11 Interdisciplinary Department of Medicine, Microbiology and Virology Unit, School of Medicine, University of Bari “Aldo Moro”, 70124 Bari, Italy; 12 School of Medicine, Phan Chau Trinh Medical University, Ho Chi Minh, 700000, Vietnam

**Keywords:** hepatitis B virus, genotype, sub-genotype, hepatocellular carcinoma

## Abstract

Untreated chronic hepatitis B virus (HBV) infection can lead to chronic liver disease and may progress to cirrhosis or hepatocellular carcinoma (HCC). HBV infection has been prevalent in Vietnam, but there is little information available on the genotypes, sub-genotypes, and mutations of HBV in patients with HBV-related HCC confirmed by histopathological diagnosis. We studied the molecular characteristics of HBV and its genetic variants in Vietnamese HCC patients after liver tumor resection. We conducted a descriptive cross-sectional study on 107 HBV-related HCC hospitalized patients from October 2018 to April 2019. The specimens collected included EDTA anticoagulant blood and liver tissues. Extracted HBV DNA was subjected to whole genome sequencing by the Sanger method. We discovered 62 individuals (57.9%) with genotype B and 45 patients (42.1%) with genotype C, with only sub-genotypes B4 and C1. Among the mutations, the double mutation, A1762T-G1764A, had the most significant frequency (73/107 samples; 68.2%) and was higher in genotype C than in genotype B (*p* < 0.001). The most common genotypes found in HCC patients in this investigation were B and C, with sub-genotypes B4 and C1 for each. The prevalence of genotype B4 was greater in HBV-infected Vietnamese HCC patients.

## 1. Introduction

The hepatitis B virus (HBV) infects two billion people, one-third of the world’s population [1]. According to the World Health Organisation (WHO), the prevalence of infection varies significantly between the geographic areas, ranging from 0.7% in America’s regions to 6% in African and Western Pacific regions [2]. HBV infection can lead to chronic conditions and progress to liver cirrhosis and hepatocellular carcinoma (HCC) [1,2]. In 2016, HBV-related deaths from cirrhosis and HCC were 365,600 and 349,500, respectively [3]. Therefore, HBV and derived HCC are major global health problems. HCC is a significant encumbrance to global health, with over 70% of cases reported in Asia [4]. The age-standardized mortality rates illustrated that Eastern Asia, Northern Africa, and Southeastern Asia are the top three regions with 16.0, 13.9, and 13.2, respectively [1]. The significant risk factors that lead to the development of HCC include hepatitis B virus, hepatitis C virus, dietary intake of aflatoxin B1, and excessive alcohol consumption [4,5]. The hepatitis B virus accounted for approximately 33–50% of the total causes, leading to 420,000 HCC cases annually [4,5]. HBV is a prototype of the family Hepadnaviridae, whose genetic material is partially double-stranded, relaxed-circular DNA (3kb in length) [6]. Unlike other DNA viruses, HBV requires reverse transcriptase for its replication [7,8]. Due to the low fidelity of reverse transcriptase during reproduction, the HBV mutation rate is approximately 1.4–3.2 × 10^−5^ base substitutions/site/year or 10^−7^ base substitutions/site/day [7,8]. Although there are high substitution rates in the HBV genome, 10 genotypes (A–J) and over 30 HBV sub-genotypes were determined based on the nucleotide sequences [9]. At least 8% of nucleotide differences between genotypes and approximately 4–8% of nucleotide differences between sub-genotypes were classified [9]. Multiple criteria for determining the HBV sub-genotypes include full-length analysis, range of nucleotide divergence, bootstrap values, recombinant strain exclusion, strain’s specific motif, and random reference sequence [10]. HBV genotyping is crucial in establishing the exact relationship between HBV genotypes and geographic distribution, clinical features, disease progression, and the response to antiviral treatment [9]. These data will allow medical scientists to configure better early detection and treatment strategies for HBV infection.

Up to now, limited studies have been conducted on HBV genotypes’ molecular characteristics in HCC patients in Vietnam. These research studies were almost exclusively done on mixed samples of patients with acute or chronic hepatitis B virus infection, cirrhosis, and liver cancer, but not solely on liver cancer patients. Even patients with liver cancer have just been diagnosed principally with AFP and diagnostic imaging. In addition, most of the studies, genotypes, and sub-genotypes of HBV have been sorted out using the partial sequence of the S gene, pre-core/core gene, or polymerase gene, instead of the entire HBV genome [11,12]. In 2015, a population-based study in Binh Thuan province showed that HBV genotype B (75.3%) was more common than genotype C (11.7%) [11]. Meanwhile, hospital-based research found that genotype B accounts for 72.6% of overall samples, compared to 27.4% of samples with genotype C [12]. In addition, the association between HBV and HCC is still poorly described in the Vietnamese population despite the high prevalence and numbers. HBV associated with HCC patients was found in only one study conducted by Toan et al. [13], which showed that 34.5% of HBV-associated HCC patients were genotype C, and 11.9% of patients carried genotype B—with the method of sequencing the core region of the HBV genome [13]. To validate the authentic HBV genotype in patients with HBV-infected liver cancer in Vietnam, we want to examine only patients with confirmed HCC by histological diagnosis and genotype and sub-genotype identification by sequencing the complete HBV genome. In line with this trend, our study investigated the molecular features of HBV and its genetic variants, focusing on HBV-infected Vietnamese HCC patients after liver tumor resection.

## 2. Materials and Methods

From October 2018 to April 2019, 179 HCC patients hospitalized in the Department of Liver Tumor at Cho Ray Hospital were recruited for this prospective research. All patients were chosen according to the existence of HBV-infected HCC. We found hepatitis B surface antigen (HBsAg) and hepatitis C viral antibody (anti-HCV) by using qualitative tests (the Cobas e601 Roche automated immune system). HBV infection was authenticated by seropositive with HBsAg. Positive blood anti-HCV test results confirmed HCV infection. Co-infection with HCV cases was excluded by seropositive with anti-HCV and RT-qPCR in liver tissue. Histopathology reports after surgical excision confirmed the HCC diagnosis for individuals. For each patient, we got 5 mL of blood and 2 liver tissue samples that were approximately 2 cm × 3 cm (normal and malignant tissues) for DNA/RNA extraction, and reconfirmed positive HBV and negative HCV infections by real-time PCR. We used the liver tissue sample routine if we could not get the complete genome in the blood sample. The research board of the local institution allowed the inquiry of Cho Ray Hospital and 108 Military Central Hospital (Approval number 3746). All participants supplied written informed consent.

### 2.1. DNA Extraction, Nested PCR, and Complete Genome Sequencing

The patient’s blood was centrifuged at 3000 rpm for 10 min to collect plasma for DNA extraction. For liver tumors: 100 mg of liver tissue homogenized in 900 uL of PBS 1X by Tissue Lyser II system (Qiagen, Hilden, Germany) at 30 Hz for 2 min, followed by centrifuging at 12,000 rpm for 3 min. We used the upper solution for DNA extraction. The extraction followed the instruction of the QIAamp DNA mini-kit. Real-time PCR confirmed HBV infection on the CFX 96 Touch Real-time PCR Detection System. HBV primers and probes targeted the HBV genome’s core region, which was designed as follows: HBc1F, 5′-AGTGTGGATTCGCACTCCT-3′; HBc1R, 5′-GAGTTCTTCTTCTAGGGGACCTG-3′; and probe HBcP1, 5′-CCAAATGCCCCTATCTTA TCAACACTTCC-3′. We amplified the complete HBV genome in overlapping fragments by nested PCR. The primers were described in Table S1 (in Supplementary Data). PCR reactions were done in 20 μL of buffer containing 1.25 U AmpliTaq Gold Polymerase (ThermoFisher Scientific, Waltham, MA, USA), 200 µM dNTPs, 2 mM MgCl_2_, 1 µLGC enhancer, and 15 pmol of primers. The thermal cycles for the first round were: 1 cycle of 95 °C-10 min; 40 cycles of 95 °C-30 s, 55 °C-45 s, and 72 °C-210 s; and 1 cycle at 72 °C for 7 min. The thermal cycles for the second round were: 1 cycle of 95 °C-10 min; 35 cycles of 95 °C-30 s, 55 °C-45 s, and 72 °C-90 s; and 1 extension cycle at 72 °C for 5 min. PCR products were checked by electrophoresis on 2% agarose. The purified step was performed by PCR Illustra ExoProStar 1-step (GE Healthcare Life Science, Buckinghamshire, UK). Sanger sequencing was performed by the BigDye Terminator v3.1 Cycle Sequencing Kit (ThermoFisher Science, Waltham, MA, USA). All sequencing data were analysed by ABI 3130 XL at Nam Khoa Biotek.

### 2.2. HCV RNA Detection

Following the directions of the QIAamp Viral RNA mini-kit, we extracted RNA from the pre-treated samples. The HCV infection was determined by quantitative Reverse Transcriptase-PCR (RT-qPCR). This research confirmed HCV infection by real-time PCR on the CFX 96 Touch real-time PCR Detection System. HCV gene-specific primers and probes were designed as follows: VGC-TqF, 5′-CCTGGAGATTTGGGCGTGC-3′;

VGC-TqR, 5′-GCAAGCACCCTATCAGGCAGT-3′;

VGC-TqP, 5′-TTGGGTCGCGAAAGGCCTTGTGGT-3′.

### 2.3. Phylogenetic Analysis

The phylogenetic analysis aligned the overlapping sequences using CLC Main Workbench 5.5, followed by multiple alignments by MUSCLE of MEGA 7.0. A phylogenetic tree constructed by the maximum likelihood method using the general time-reversible (GTR) model plus gamma distributed with Invariant sites (GTR + G + I). Bootstrap re-sampling was carried out 100 times to confirm the reliability of the phylogenetic tree topologies. The reference genotypes and sub-genotypes were obtained from GenBank and described in Table S2 (in Supplementary Data).

### 2.4. Mutational Analysis

The HBV preS1/S2/S region was screened for mutations as described earlier: preS1/S2 deletion, preS1 mutations (A2962G, C2964A, C3026A/T, C3116T), preS2 mutations (T31C, T53C), S mutations (N3S, T116 N, P120S/T, I/T126S/A, Q129H/R, M133 L/T, K141E, P142S, D144E, G145R, V184A, and S204R) [12]. Mutations in the X regions/Enhancer II/Basal core promoter (BCP) analyzed: G1613A, C1653T, G1721A, A1762T, G1764A/C, C1766T, T1768A, A1775G [12,14,15] (Table 1). In the pre-core/core (PC/C) regions, following mutations screened: T1858C, G1896A, G1899A, C2002T, A2149T/C, A2159G, A2189T/C, C2191T, C2198A, G2203A [12,15,16]. In the reverse transcriptase (RT) region, primary and secondary drug mutations screened (rt80, rt169, rt173, rt180, rt181, rt184, rt194, rt202, rt204, rt236 and rt250), putative nucleos(t)ide analogue resistance (NAr) mutations (rt53, rt54, rt82, rt84, rt85, rt91, rt126, rt128, rt139, rt153, rt166, rt191, rt200, rt207, rt213, rt214, rt215, rt217, rt218, rt221, rt229, rt233, rt237, rt238, rt245, and rt256), and pre-treatment mutations (rt38, rt124, rt134, rt139, rt224 and rt242) [12,17].

### 2.5. Statistical Analysis

The data were analyzed with SPSS 20 (version 2020). A Pearson Chi-Square and Fisher’s exact test were used to compare the categorical variables. A *p*-value < 0.05 was considered a statistically significant difference.

## 3. Results

### 3.1. Phylogenetic Analysis of HBV Genotype

**Figure 1:**
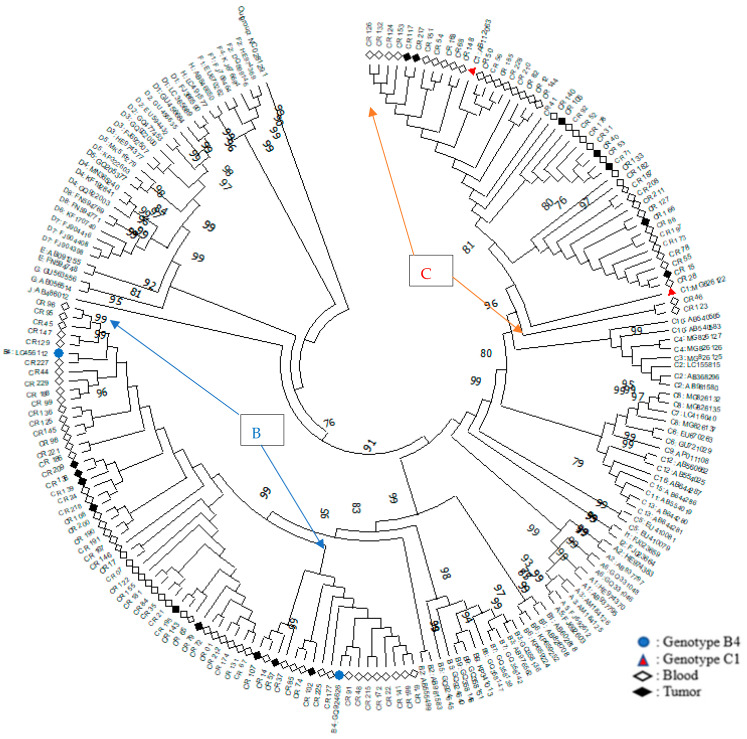
Phylogenetic tree of 107 HBV samples with reference sequences of each genotype (A–J) and their corresponding sub-genotype.

The phylogenetic analysis included 107 patients in the study whose complete genome sequences were obtained and the maximum likelihood method was applied for the construction of the phylogenetic tree (our team was able to sequence 107 samples in this investigation effectively. A lack of amplification was due to the low viral load and the presence of inhibitors in the liver tissue samples. As a result, we could not get HBV sequences for the remaining 72 specimens). (Figure 1). We had 62 patients (57.9%) diagnosed with genotype B, which was more than genotype C-45 patients (42.1%) (Figure 1 & Table 2). The phylogenetic analysis illustrated that genotype B (sub-genotype B4) and C (sub-genotype C1) were the primary isolates among ten genotypes discovered worldwide. Of 62 sequences defined as genotype B, 53 (85.5%) originated from blood.

Meanwhile, among 45 sequences of genotype C (84.44%), 38 of them were from blood (84.4%) (Figure 1 & Table 2). They constructed the tree using the maximum likelihood method with GTR + G + I. The number of bootstrap replications was 100. Each reference sequence began with a letter of the corresponding sub-genotype, followed by a GenBank accession number. The reference genotypes for B4 are highlighted with a filled blue circle and genotypes C1 are highlighted with a filled red triangle; the sequences obtained from blood are highlighted with a white diamond shape, and the sequences obtained from tissue are highlighted with a dark diamond shape.

### 3.2. Distribution of Age and Gender Factors by Genotypes

Regarding age, the mean value of patients diagnosed with genotype B was 52.9 years old, with over 50% of people belonging to the 41–60 age group. Meanwhile, 49.4 years old was the mean value of genotype C patients, with 24.4% in the 31–40 and 51–60 age groups and 33.3% in the 41–50 age group. Regarding gender in the study, the ratio of men to women was 7.2:1.

### 3.3. Mutational Regions of HBV Genotypes B and C

There were 18 isolates observed with a preS1/S2/S gene mutation. The nucleotide substitutions, such as A2962G, C2964A, and T766A, were only observed in genotype B (*p* < 0.002). The A162G and C531T were more frequent in genotype C (*p* < 0.001). We discovered C3026A in 61 of 62 isolates for genotype B and 44 out of 45 for genotype C (*p* = 0.818) (Table 3). On gene X, mutations at locations G1721A, A1762T, G1764A, A1775G, and double mutations at A1762T + G1764A were statistically more prevalent in HBV genotype C than in genotype B (*p* < 0.001) (Table 3). We detected A1762T + G1764A double mutations in 68.22% (73 out of 107) patients. The pre-core/core gene mutation, T1858C, was widespread in patients with genotype C (41 out of 45) (*p* = 0.000). The G1896A appeared with statistical significance in genotype B (46 out of 62) (*p* = 0.000) (Table 3). For mutations in the polymerase gene, the amino acid substitutions, including S53N/D, V207M, S213T, F221Y, N238H, and S256G, were significantly predominant in genotype B when compared to genotype C (*p* < 0.05). Only N238T was correlated with genotype C (*p* < 0.001). In the pre-treatment mutation, Y124H and I224V were more frequent in genotype B (*p* < 0.001) (Table 3).

## 4. Discussion

It is known that the infectious process, clinical symptoms, and responsiveness to HBV treatment highly depends on the virus’ genotype and sub-genotype [18]. Therefore, HBV sub-genotyping was crucial in planning and implementing community-based health strategies. In Vietnam, studies on HBV in patients with HCC remain insufficient; the absence of an appropriately robust, standardized genotyping procedure led to report discrepancies. Using polymerase chain reaction-restriction fragment length polymorphisms (RFLP), partial gene sequencing on S or pre-core/core promoter regions, and whole-genome sequencing requires a cautious procedure and accurate interpretation [10,12,13,19].

Despite several advances in the HBV classification method, the phylogenetic analysis of the entire HBV genome remains the “gold-standard” approach [10].

The collected data showed that genotypes B and C were the two prevalent genotypes in Vietnam. This study has been performed by the Sanger sequencing method for HBV’s whole-genome sequencing to get reliable results. Although our findings aligned with several publications, the proportion between genotypes B and C differed [11,12,19]. In the 2015 and 2017 reports, the ratio between HBV genotype B vs. C was quite impressive, 75.3:11.7 and 72.6:27.4 [11,12]. In a study by Huy et al. in 2004 on HBV-associated HCC samples, the proportions between genotype B vs. C were closer to 57.9:42.1 and 52.1:47.9, respectively [19]. Though all previous studies were performed on different specimen types, these values still confirmed the higher prevalence of genotype B compared to C in Vietnam. This observation was also reported in studies completed in Taiwan, Japan, and Thailand [20,21,22]. Another aspect was the addition of other genotypes besides B and C. In 2006, the study in Ha Noi using the RFLP technique showed the presence of genotypes A–G [13]. The survey conducted in Binh Thuan province using enzyme immunoassay and direct sequencing revealed genotype D (5.2%) [11]. The differences in the genotype B:C proportion and other genotypes appear to be related to the examined individuals’ different criteria, the sampling region, and the molecular-based methodologies. Eventually, these data lacked accuracy and validity without robust sequencing and a phylogenetic method.

Here, we could further classify the samples into sub-genotype based on the phylogenetic analysis, including 57.9% B4 and 42.1% C1. Bui et al. also mentioned the prevalence of B4 and C1 in 2007 [12]. Therefore, B4 and C1 were considered the predominant sub-genotype in Vietnam. Regarding age, the mean value of the study subjects was 51.4 ± 11.4, which was considered the youngest age of HCC patients compared to 54.4 ± 12.9 in Thailand and 55.1 ± 10.8 in Japan [20,21,22]. In Thailand, the mean age of genotype B patients (61.1 ± 9.8) was older than genotype C patients (51.3 ± 13.1) (*p* = 0.001) [22]. In contrast, genotype B patients (50 ± 14) were significantly younger than genotype C patients (59 ± 10) in Taiwan (*p* = 0.006) [20]. These differences may be because of the sample size and geographical location. Therefore, the relation between age and the HBV sub-genotype needs further investigation. Regarding gender, eight-times as many male patients took part in the study as females did. The results agreed with several publications with a male:female ratio ranging from 3:1 in Japan to 6.7:1 in Thailand [21,22]. The differential distribution relates to combining several factors or hormones, estrogen, and androgen, during tumorigenesis [4,23]. However, the available epidemiological evidence was still limited and remained controversial.

Detailed mutational analyses were conducted in our study, which shed light on the key HBV genome regions and mutations of interest. In a longitudinal study of HCC patients, pre-S deletion and pre-S2 start codon mutation were shown to be independently related to HCC [24]. These two types of mutations weren’t observed at initial infection but would appear during disease progression [24]. Several studies reported that the preS1/S2 region mutation is associated with HCC [7,25,26]. An attempt to develop a vaccine based on the preS1 region was also conducted [25]. About the S gene covering the “a” determinant region (amino acid 124–147), mutations in this area would alter the HBsAg antigenicity, leading to the prevention of HBsAg detection or vaccine evasion [7]. With some statistically significant mutations being found in the preS1/ S2/S region (A2962G, C2964A, A162G, C531T, T766A) (*p* < 0.001), further research on the HBV developmental stage or HCC detection marker in this region should be conducted, as well as the X-ORF-encoded HBx, which played vital roles in transcription, signal transduction, and apoptosis [7]. Multiple studies illustrated the significant association between X mutation and HCC development, especially the double mutation, A1762T/G1764A, associated with genotype C [7].

In our study, double mutation A1762T/G1764A appeared in genotype C more frequently than in B (43/45 samples—95% for genotype C—and 31/62 samples—50% for genotype B). A study conducted in 2017 also supported the dominance of double mutations in genotype C [12]. The mutation, G1721A/A1775G/T1858C, was significantly associated with genotype C [15]. Our study was in line with the results of Huang et al. [15], though it is required to work with more robust clinical data to assess further these combined mutations (G1721A/A1775G/T1858C and A1762T/G1764A). In the pre-core/core region, G1896A was predominant in genotype B (46/62 samples—74.2%) compared to C (2/45 models—4.44%). The result supported the finding of Huang et al. in 2015 with a 58.9% mutation rate in genotype B compared to 5.4% in C [15]. G1896A is responsible for the PC’s premature stop codon at position 28 [7,27]. Consequently, HBeAg translation was completed, and negative HBeAg was detected [27]. In terms of polymerase mutations, our findings supported the idea of the absence of primary or secondary drug resistance mutations [12,17]. Six of the eight sites of genotype-dependent AA-polymorphisms found in 107 sequences in this study’s scale had previously been reported in Vietnam and China on chronic and treatment-naive HBV samples, respectively [12,17].

The other two sites, V207M and S213T mutation, appeared to be dominant in genotype B compared to C. The detection of the remaining two areas might be because of the type from the initial sample (chronic vs. HCC), the sampling location, or the number of sequences analyzed. This study did not investigate the correlation between risk factors, clinical presentations, and HBV genotype. In addition, only HBV associated with the HCC sample was obtained; HBV mutations related to disease progression were not documented. These investigations, coupled with the findings in this study, will improve our understanding of the relationship between HBV genotypes and disease progression in Vietnam, which has highly prevalent HBV infection [28,29].

Our study had some limitations: it was a single-center study. We sequenced the complete HBV genome, but the sample size was not large enough to reflect the national or continental situation.

## 5. Conclusions

Although HBV genotypes B and C are predominant within the Vietnamese population, HBV genotype B is more prevalent than C. Vietnam has only detected sub-genotypes B4 and C1. Regarding HBV variations with statistical significance, we see five mutations in the S region (A2962G; C2964A; A162G; C531T; T766A), three mutations in the X region (G1721A; A1762T + G1764A; A1775G), two mutations in the pre-core/core region (T1858C; G1896A), and nine protein substitutions in the polymerase region (S53N, V207M, S213T, F221Y, N238H, N238T, S256G, Y124H, I224V). These data provide a better knowledge of the molecular biology characteristics in HBV-infected HCC patients in Vietnam.

## Figures and Tables

**Table 1:** Analysis of polymerase gene mutation.

Region		Nucleotide	Amino Acid	Genotype B *n*= 62	Genotype C *n* = 45	*p* Value
PreS1 (2848–3204)		A2962G	N38E	60 (96.8)	0	<0.001 *
		C2964A	N38E	60 (96.8)	0	<0.001 *
		C3026A	A60V	61 (98.4)	44 (97.8)	0.818 *
		C3116T	A90V	2 (3.2)	1 (2.2)	0.756 *
PreS2 (3205–155)		A3205G	M1V (Stop)	5 (8.1)	2 (4.4)	0.696 *
		A3205C	M1L (Stop)	1 (1.6)	0	1.000 *
		T3206C	M1T	0	3 (6.7)	0.071 *
		T31C	No change	1 (1.6)	0	1.000 *
		T53C	F22I	9 (14.5)	11 (24.4)	0.193 **
S (155–835)		A162G	N3S	1 (1.6)	45 (100)	<0.001 *
		C512T	P120S	2 (3.2)	0	0.508 *
		C512A	P120T	5 (8.1)	0	0.072 *
		C531T	T126I	0	25 (55.6)	<0.001 *
		C531A	T126N	0	2 (4.4)	0.175 *
		A551T	M133L	5 (8.1)	0	0.072 *
		T552C	M133T	0	1 (2.2)	0.421 *
		G587A	G145R	0	1 (2.2)	0.421 *
		G588C	G145A	0	2 (4.4)	0.175 *
		T705C	V184A	3 (4.8)	1 (2.2)	0.637 *
		T766A	S204R	11 (17.7)	0	0.002 *
X (1374–1838) Enhancer II (1685–1773)		G1613A	NC	23 (37.1)	16 (35.6)	0.870 **
		C1653T	H94Y	8 (12.9)	6 (13.3)	0.948 **
		G1721A	No change	3 (4.8)	39 (86.7)	<0.001 **
X (1374–1838) BCP (1751–1769)		A1762T	K130M	32 (51.6)	43 (95.6)	0.000 *
		G1764A	V131I	31 (50)	43 (95.6)	0.000 *
		G1764C		0	1 (2.2)	0.421 *
		A1762T + G1764A		31 (50)	42 (93.3)	<0.001 *
		C1766T + (G1764A)	V131I	6 (9.9)	3 (6.7)	0.731 *
		C1766T	No chance	1 (1.6)	0	1.000 *
		T1768A	F132Y	8 (12.9)	4 (8.9)	0.758 *
		A1775G	No change	1 (1.6)	24 (53.3)	0 *
Precore (1814–1900)		T1858C	No change	1 (1.6)	41 (91.1)	<0.001 *
		G1896A	W28Stop	46 (74.2)	2 (4.4)	<0.001 *
		G1899A	G29D	6 (9.7)	4 (8.9)	1.000 *
Core region (1901–2452)		C2002T	No change	1 (1.6)	1 (2.2)	1.000 *
		A2149T/C	E83D	5 (8.1)	3 (6.7)	1.000 *
		A2159G		11 (17.7)	6 (13.3)	0.538 **
		A2189T	I97F	2 (3.2)	1 (2.2)	1.000 *
		A2189C–C2191T				
		A2189C	I97L	19 (30.6)	15 (33.3)	0.768 **
		A2189T-C2191T				
		C2198A	L100I	15 (24.2)	10 (22.2)	0.812 **
		G2203A	No change	1 (1.6)	0	1.000 *
Polymerase (2307–1623)	Putative Nar mutation	G287A	S53N	47 (75.8)	0	<0.001 *
		A286G-G287A	S53D	7 (11.3)	0	0.021 *
		T382G	S85A	0	1 (2.2)	0.421 *
		A400C	I91L	56 (90.3)	40 (88.9)	0.810 **
		A400C-T402G				
		C505T	H126Y	1 (1.6)	0	1.000 *
		C512A	T128N	5 (8.1)	0	0.072 *
		C512T	T128I	1 (1.6)	0	1.000 *
		A511G	T128A	0	1 (2.2)	0.421 *
		A544C	N139H	1 (1.6)	0	1.000 *
		A544C-C546A	N139Q	1 (1.6)	0	1.000 *
		A544G-C546A	N139E	0	1 (2.2)	0.421 *
		G587A	R153Q	0	2 (4.4)	0.175 *
		G700A	V191I	0	1 (2.2)	0.421 *
		G748A	V207M	11 (17.7)	0	0.002 *
		G748C	V207L	0	1 (2.2)	0.421 *
		T766A	S213T	10 (16.1)	0	0.005 *
		A774C	Q215H	2 (3.2)	0	0.508 *
		T779G	L217R	1 (1.6)	0	1.000 *
		A/G783C	E218D	2 (3.2)	0	0.508 *
		T791A	F221Y	59 (95.2)	5 (11.1)	<0.001 *
		T790C-T791A	F221H	1 (1.6)	0	1.000 *
		T814A	L229M	2 (3.2)	0	0.508 *
		T814G	L229V	1 (1.6)	0	1.000 *
		T815G	L229W	0	1 (2.2)	0.421 *
		A826G	I233V	2 (3.2)	0	0.508 *
		A841C-T843C	N238H	62 (100)	0	<0.001 *
		A841C-A842C				
		A842C	N238T	0	11 (24.4)	<0.001 *
		A841G-A842C	N238A	0	3 (6.7)	0.071 *
		A895G	S256G	12 (19.4)	0	0.001 *
		A895G-G896C	S256A	4 (6.5)	0	0.137 *
	Pretreatment mutation	A241G	T38A	0	1 (2.2)	0.421 *
		C242A	T38K	0	1 (2.2)	0.421 *
		T499C	Y124H	61 (98.4)	4 (8.9)	<0.001 *
		T499A	Y124N	1 (1.6)	0	1.000 *
		T499G	Y124D	0	1 (2.2)	0.421 *
		C/T531G	D134E	0	3 (6.7)	0.071 *
		G529A-A530G	D134S	0	1 (2.2)	0.421 *
		G529A	D134N	1 (1.6)	1 (2.2)	0.818 *
		A544C-C546A	N139Q	1 (1.6)	0	1.000 *
		A544G-C546A	N139E	0	1 (2.2)	0.421 *
		A544C	N139H	2 (3.2)	0	0.508 *
		A799G	I224V	61 (98.4)	28 (62.2)	<0.001 *

Note: * Fisher’s Exact test, ** Pearson Chi-Square.

**Table 2:** Distribution of patient characteristics with HBV infection.

	Total *n* (%)	Genotype B *n* (%)	Genotype C *n* (%)
	107 (100%)	62 (57.9%)	45 (42.1%)
**Subgenotype**			
B4	62 (57.9%)		
C1	45 (42.1%)		
**Age (Mean ± SD)**	51.4 ± 11.4	52.9 ± 11.9	49.4 ± 10.4
≤30	1 (0.9%)	1 (1.6%)	0
31–40	20 (18.7%)	9 (14.5%)	11 (24.4%)
41–50	35 (32.7%)	20 (32.3%)	15 (33.3%)
51–60	27 (25.2%)	15 (24.2%)	12 (26.7%)
61–70	17 (15.9%)	12 (19.4%)	5 (11.1%)
71–80	7 (6.5%)	5 (8.1%)	2 (4.4%)
**Gender**			
Male	94 (87.9%)	55 (88.7%)	39 (86.7%)
Female	13 (12.2%)	7 (11.3%)	6 (13.3%)
**Biochemistry (Mean ± SD)**			
ALT (U/L)	56.8 ± 47.6	45.2 ± 39.3	72.9 ± 53.4
AST (U/L)	61.9 ± 40.4	61.1 ± 46.1	62.9 ± 31.3
Bilirubin (mg/dL)	0.8 ± 0.6	0.8 ± 0.3	0.8 ± 0.9
GGT (U/L)	151.4 ± 122.6	143.6 ± 114.8	162.1 ± 113.2
Albumin (g/dL)	4.0 ± 0.4	4.0 ± 0.3	4.0 ± 0.4
PT (s)	13.5 ± 0.7	13.5 ± 0.7	13.5 ± 0.7
Hb (g/L)	139.3 ± 27.3	138.8 ± 24.0	140.0 ± 31.6
WBC (G/L)	10.0 ± 15.9	10.0 ± 16.2	10.0 ± 15.7
Platelet (G/L)	230.5 ± 74.4	243.2 ± 77.9	213.0 ± 66.2

**Table 3:** Summary of HBV mutation.

Region	Nucleotide	Amino Acid	Genotype B *n*= 62	Genotype C *n* = 45	*p* Value
PreS1 (2848–3204)	A2962G	N38E	60 (96.8)	0	<0.001 *
	C2964A	N38E	60 (96.8)	0	<0.001 *
	C3026A	A60V	61 (98.4)	44 (97.8)	0.818 *
S (155–835)	A162G	N3S	1 (1.6)	45 (100)	<0.001 *
	C531T	T126I	0	25 (55.6)	<0.001 *
	T766A	S204R	11 (17.7)	0	0.002 *
X (1374–1838) Enhancer II (1685–773)	G1721A	No change	3 (4.8)	39 (86.7)	<0.001 **
X (1374–1838) BCP (1751–1769)	A1762T	K130M	32 (51.6)	43 (95.6)	<0.001 *
	G1764A	V131I	31 (50)	43 (95.6)	<0.001 *
	A1762T + G1764A		31 (50)	42 (93.3)	<0.001 *
	A1775G	No change	1 (1.6)	24 (53.3)	<0.001 *
Precore (1814–1900)	T1858C	No change	1 (1.6)	41 (91.1)	<0.001 *
	G1896A	W28Stop	46 (74.2)	2 (4.4)	0.000 *
Polymerase (2307–1623) Putative Nar mutation	G287A	S53N	47 (75.8)	0	<0.001 *
	A286G-G287A	S53D	7 (11.3)	0	0.021 *
	G748A	V207M	11 (17.7)	0	0.002 *
	T766A	S213T	10 (16.1)	0	0.005 *
	T791A	F221Y	59 (95.2)	5 (11.1)	<0.001 *
	A841C-T843C	N238H	62 (100)	0	<0.001 *
	A841C-A842C	N238T	0	11 (24.4)	<0.001 *
	A842C				
	A895G	S256G	12 (19.4)	0	0.001 *
Polymerase (2307–1623) Pretreatment mutation	T499C	Y124H	61 (98.4)	4 (8.9)	<0.001 *
	A799G	I224V	61 (98.4)	28 (62.2)	<0.001 *

Note: * Fisher’s Exact test, ** Pearson Chi-Square.

## Data Availability

The data presented in this study are available on request from the corresponding author. The data are not publicly available due to local law limitations.

## Supplementary Materials

The following supporting information can be downloaded at: https://www.mdpi.com/article/10.3390/diagnostics12102393/s1, Table S1: Primers used for complete genome DNA amplification and sequencing; Table S2: GenBank accession numbers of reference sequences of HBV genotype.

## Abbreviations

HBV	Hepatitis B Virus
WHO	World Health Organization
HCC	Hepatocellular Carcinoma
RFLP	Restriction Fragment Length Polymorphism reaction
