# Supplementary material for: Characteristics of Hepatitis B Virus Genotype and Sub-Genotype in Hepatocellular Cancer Patients in Vietnam
Source: Diagnostics (Basel). 2022 Oct 1;12(10):2393. doi: 10.3390/diagnostics12102393 (PMC9600587; doi:10.3390/diagnostics12102393)
Supplement: Supplementary file 1 [file diagnostics-12-02393-s001.zip › diagnostics-1901846-supplementary.pdf]

Supplementary data

Table S1: Primers used for complete genome DNA amplification and sequencing

	Primer	Sequence (5′-3′)	Position	% GC	Tm (°C)	Size (bp)
First round	HBV1 (a)	CCGGAAGAATTCTTTTTCACCTCTGCCTAATCA	1820 -1841	42	55	3215
	HBV4R	CCGGAGAGCTCATGCTCTTCAAAAGTTGCATGGT GCTGGTG	1824 - 1804	54	60	
A	HBV1 (a)	CCGGAAGAATTCTTTTTCACCTCTGCCTAATCA	1820 - 1841	42	55	497
	BG1R (a)	ATAGGGGCATTTGGTGGTCT	2316 - 2297	50	58	
B	HBC1 (a)	AGTGTGGATTCGCACTCCT	2269 - 2287	53	59	828
	PS8R (a)	ARGCCCTGAGCCTGAGGGCTC	3096 - 3078	69	60	
C	P1 (a)	TCACCATATTCTTGGGAACAAGA	2817 - 2839	39	55	1103
	S1-2 (a)	CGAACCACTGAACAAATGGC	704 - 685	50	56	
F	251F (b)	GACTYGTGGTGGACTTCTC	251 - 269	55	56	940
	1190R (b)	TCAGCAAAYACTYGGCA	1190 - 1174	47	53	
G	595F (b)	CACHTGTATTCCCATCCCA	595 - 613	47	53	1231
	HBV2 (a)	CCGGAAGAATTCAAAAAGTTGCATGGTGCTGG	1825 - 1806	47	58	
E	HBx1	GTCCCCTTCTTCATCTGCCGT	1487 – 1507	57	61	339
	HBV2 (a)	CCGGAAGAATTCAAAAAGTTGCATGGTGCTGG	1825 - 1806	47	58	

Note: Primers (a) are from Huy TTT et al. (2004) [19]; Primers (b) are from Wang Y et al. (2021) [28].

Table S2: GenBank accession numbers of reference sequences of HBV genotype.

HBV Genotype	Sub-genotype	Accession number	Nationality
A	A1	HE974370	France
		AB937795	Japan
	A2	HE974383	France
		AB937797	Japan
	A3	AM184126	France
		AM184125	France
	A5	FJ692612	Haiti
		FJ692603	Haiti
	A6	GQ331048	Africa
		GQ331046	Africa
B	B1	AB602818	Japan
		AB828708	Japan
	B2	AB981583	Japan
		AB555499	Taiwan
	B3	GQ358136	Indonesia
		AB976562	Indonesia
	B4	GQ924626	Malaysia
		LC456112	Cambodia
	B5	GQ924640	Malaysia
		GQ924645	Malaysia
	B6	KP659252	Canada
		KP659224	USA
	B7	GQ358142	Indonesia
		GQ358139	Indonesia
	B8	GQ358147	Indonesia
	B9	GQ358151	Indonesia
		GQ358148	Indonesia
		KP341013	Indonesia
C	C1	MG826122	China
		AB112063	Viet Nam
	C2	AB368296	Japan
		AB981580	Japan
		LC155815	Japan
	C3	MG826125	China
	C4	MG826127	China
		MG826126	China
	C5	EU410081	Philippine

		EU410079	Philippine
	C6	EU670263	Philippine
		GU721029	South Korea
	C7	LC416040	Indonesia
	C8	MG826137	China
		MG826132	China
		MG826135	China
	C9	AP011108	Indonesia
	C10	AB540583	Indonesia
		AB540585	Indonesia
	C11	AB554019	Indonesia
	C12	*AB560662*	Indonesia
		AB554025	Indonesia
	C13	AB644280	Indonesia
		AB644281	Indonesia
	C15	AB644286	Indonesia
	C16	AB644287	Indonesia
D	D1	GU456684	Iran
		LC365689	Japan
		FJ386590	China
	D2	GQ477453	Poland
		EU594432	Russia
		GU456635	Iran
	D3	HE974377	FRANCE
		GQ922000	Canada
		FJ692507	Haiti
	D4	KF192841	India
		MN365240	Russia
		GQ922003	Canada
	D5	GQ205377	India
		KP322603	India
		MK516279	India
	D6	KF170740	Sudan
	D7	FJ904416	Tunisia
		FJ904398	Tunisia
		FJ904408	Tunisia
	D8	FN594769	Niger
		FN594771	Niger
E		FN594748	Niger

		AB091255	Japan
F	F1	EU670262	Peru
		FJ709464	Chile
	F2	DQ899146	Venezuela
		HE974369	Martinique
	F4	KJ676694	Argentina
G		AB056514	Japan
		GU563556	Belgium
H		LC491577	Japan
		AB846650	Japan
I	I1	FJ023659	Laos
	I2	FJ023664	Laos
J		AB486012	Japan
Outgroup<br>Wolly Monkey		NC028129	
